# Trait anxiety affects attentional bias to emotional stimuli across time: A growth curve analysis

**DOI:** 10.3389/fnins.2022.972892

**Published:** 2022-09-14

**Authors:** Chen Xing, Yajuan Zhang, Hongliang Lu, Xia Zhu, Danmin Miao

**Affiliations:** Military Medical Psychology School, Fourth Military Medical University, Xi'an, China

**Keywords:** trait anxiety, attention, cognitive bias, information processing, eye movements, growth curve analysis

## Abstract

Many studies have illustrated the close relationship between anxiety disorders and attentional functioning, but the relationship between trait anxiety and attentional bias remains controversial. This study examines the effect of trait anxiety on the time course of attention to emotional stimuli using materials from the International Affective Picture System. Participants with high vs. low trait anxiety (HTA vs. LTA) viewed four categories of pictures simultaneously: dysphoric, threatening, positive, and neutral. Their eye-movements for each emotional stimulus were recorded for static and dynamic analysis. Data were analyzed using a mixed linear model and growth curve analysis. Specifically, the HTA group showed a greater tendency to avoid threatening stimuli and more pupil diameter variation in the early period of stimulus presentation (0–7.9 s). The HTA group also showed a stronger attentional bias toward positive and dysphoric stimuli in the middle and late period of stimulus presentation (7.9–30 s). These results suggest that trait anxiety has a significant temporal effect on attention to emotional stimuli, and that this effect mainly manifests after 7 s. In finding stronger attentional avoidance of threatening stimuli and more changes in neural activity, as well as a stronger attentional bias toward positive stimuli, this study provides novel insights on the relationship between trait anxiety and selective attention.

## Introduction

Anxiety disorders are the most prevalent mental disorders, accompanied by a high medical burden (Wittchen et al., 2011; Kessler et al., 2012; Chisholm et al., 2016). Recent studies have shown that of the many health problems brought by the COVID-19 pandemic, anxiety disorders are the most frequent (Choi et al., 2020; Salari et al., 2020; Kwong et al., 2021).

Many studies have also shown that anxiety is very closely related to attentional function (Dalgleish and Watts, 1990; Shechner et al., 2012). Researchers have suggested that the relationship between attentional bias and anxiety should be described as bidirectional, maintained, or mutually reinforcing (Van Bockstaele et al., 2014). Reactive attention biases intensify a heightened state of anxiety, posing the risk of developing psychopathology (White et al., 2009). Based on such insights, researchers have found that training individuals to shift their prioritizing from threats to sources of safety may be a useful treatment to reduce the risk of developing anxiety disorders (Hallion and Ruscio, 2011). However, controversy persists about the effectiveness of cognitive bias modification (CBM). Cristea et al. (2015) suggest that any effect of CBM on mental health problems is likely to be small and not clinically relevant. Conversely, some meta-analyses have indicated that CBM has a small but stable mitigating effect on anxiety disorders (Krebs et al., 2018; Fodor et al., 2020). It has also been argued that CBM has positive efficacy in mitigating anxiety symptoms and that future research should focus on (1) developing procedures that more reliably induce bias change and (2) identifying the most effective clinical applications (Jones and Sharpe, 2017). Uncertainty on the effectiveness of CBM may reflect the need for further research on the relationship between anxiety and attention.

Although anxiety is especially common, its conceptual structure is relatively complicated. Spielberger (1966) suggested conceiving anxiety as multifaceted by distinguishing trait anxiety from state anxiety. Since the mid-1960s, the trait–state distinction has received wide recognition in the psychological literature (Endler, 1982, 1997; Dreger, 1985; Spielberger, 1985). Further research on state–trait anxiety revealed a double dissociation: whereas trait anxiety was related to deficiencies in the executive control network, state anxiety was associated with over-functioning of the alerting and orienting networks (Pacheco-Unguetti et al., 2010). In a study of the performance of students with ADHD in a selective attention task, the combined ADHD subtype exhibited higher trait anxiety whereas the inattentive ADHD subtype showed more state anxiety (González-Castro et al., 2015). These results suggest that trait anxiety and state anxiety significantly differ in their effects on attention. Therefore, when exploring how anxiety affects attention, it is necessary to investigate trait anxiety differently from state anxiety. Prior results also suggest the necessity for detailed study of anxiety and attention. However, traditional methods of attentional bias research based on response time have low reliability and cannot effectively measure the process of change in attention (Waechter and Stolz, 2015).

Eye-tracking technology enables more direct and reliable study of attentional bias (Marks et al., 2014; Skinner et al., 2018; Sears et al., 2019). Attentional bias studies using eye-tracking techniques have shown a significant effect of anxiety on attention to threatening stimuli (Armstrong and Olatunji, 2012; Shechner et al., 2013; Liang et al., 2017). However, this attention bias has mostly been found for state anxiety, and not for trait anxiety (Berggren et al., 2012; Quigley et al., 2012; Nelson et al., 2015). Conversely, neuroimaging studies have shown that trait anxiety is linked to impoverished recruitment of prefrontal attentional control mechanisms (Bishop, 2009; Baur et al., 2013; Modi et al., 2015). Many studies have shown that trait anxiety is a good predictor of state anxiety in different situations (William Li and Lopez, 2005; Horikawa and Yagi, 2012; Xie and Karan, 2019). Moreover, trait anxiety has higher longitudinal stability than state anxiety (Usala and Hertzog, 1991; Hong, 1998), and thus has more long-term value for predicting individual anxiety levels. To further explore the relationship between trait anxiety and attention, a more detailed analytical approach than static eye-movement data analysis may be required. We expect that eye-tracking analysis based on time-course data can facilitate detailed investigation of the process of attentional change.

Prior studies have found a significant temporal effect of attentional bias for different emotional stimuli (Kellough et al., 2008), including among depressed patients (Arndt et al., 2014). However, most previous time-series studies have used overly long time intervals that compress large amounts of temporal information. There have also been relatively few time-series studies of the relationship between trait anxiety and selective attention.

Previous studies have shown the need for more detailed examination of the effects of trait anxiety on selective attention. Time-series analysis based on eye movement data has high potential for enhancing understanding of this relationship, yet few prior studies have deployed this approach. This paper examines the relationship between trait anxiety and attention to emotional stimuli using time-course analysis, thereby addressing some gaps in the literature. Our study design is similar to that of Kellough et al. (2008): participants were presented with four types of stimuli simultaneously—dysphoric, threatening, positive, and neutral—and allowed to freely view the stimuli for 30 s. To explain the different results of previous studies on attention, we argue that the effect of trait anxiety on attention to emotional stimuli is dynamic and inconsistent over time. Therefore, this study analyzes time-course variation in attentional processes, using a mixed linear model and growth curve analysis (GCA) with 100 ms time bins to maximize the retention of valid information.

## Materials and methods

### Participants

Participants were recruited from two undergraduate years at the same university. A total of 198 participants validly completed the State Trait Anxiety Inventory—Trait scale (STAI-T; Spielberger, 1985). According to previous studies (Stegmann et al., 2019; Qi et al., 2020), we classified subjects with STAI-T scores <33 (bottom quartile) and >44 (top quartile) as the low trait anxiety (LTA) group and high trait anxiety (HTA) group, respectively. After excluding people with abnormal vision, diagnosed mental disorders, or a history of short-term psychotropic substance use, both groups participated in an eye-tracking experiment. Participants in the experiment were paid a small reward. Complete data for formal analysis were collected from 86 individuals (53 females, *M_ag*e = 20.1 years).

### Materials

The STAI-T (Spielberger, 1985) was designed to measure a stable propensity to experience anxiety and tendencies to perceive stressful situations as threatening. It comprises 20 statements requiring individuals to rate how they generally feel on a four-point scale (total score range: 20–80). The test–retest reliability coefficient is high, ranging from 0.73 to 0.86 (Spielberger, 1983). In this study, the STAI-T demonstrated good internal consistency (Cronbach's α = 0.93, *n* = 198). Average STAI-T scores were 50.4 for the HTA group (*SD* = 7.6, *n* = 46) and 29.2 for the LTA group (*SD* = 3.8, *n* = 40).

The emotional stimuli for the experiment were sourced from the International Affective Picture System (IAPS, Lang et al., 2008)—a large set of emotionally rich, internationally accessible color photographs. Each image has corresponding sentiment ratings, and the IAPS includes content across a wide range of semantic categories. This study adopts the classification of stimuli used by Kellough et al. (2008), selecting 48 images divided into four categories: dysphoric, threatening, positive, and neutral (Supplementary Table S1).

### Eye-tracking

The Eyelink 1000 Plus was selected as the eye-tracking device for this study. We set the eye-tracking sampling rate to 500 Hz. A 9-point calibration procedure was performed before each experiment. The monitor size was 17 in (37.5 × 30.0 cm), and the resolution was set to 1,024 × 768 pixels. Participants used a chin rest for stabilization control, and the distance between their eyes and the center of the monitor was 62 cm. Therefore, participants' visual angle was 32.2° × 26.0°. To ensure the pupil-diameter data were reliable, we strictly controlled the laboratory environment in terms of monitor brightness, room brightness, curtain shading, and noise.

**Figure 1:**
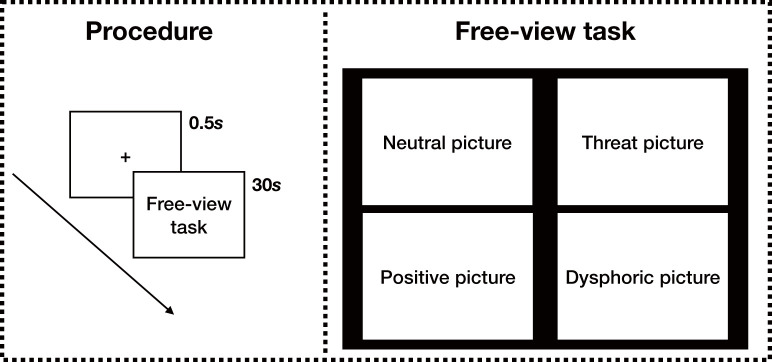
Eye-tracking experiment procedure and free-viewing task demonstration.

The free-viewing task comprised a total of 12 trials: each trial began by displaying a fixation cross for 500 ms, followed by the set of four pictures for 30 s. The fixation cross was black (25 × 25 pixels) and displayed on a gray background. During the free-viewing period, participants were asked to take a free viewing without a specific task. Four pictures of equal size were displayed in the four corners of the screen, each representing one of the four categories. The type and position of the pictures were balanced on the screen (see Figure 1), and each picture was presented only once in the experiment. The experiment duration was about 7 min. Each participant was informed about the procedure and content of the experiment through instructions given before it began.

The main eye-tracking metrics used in this study are the gaze proportion on different emotional stimuli and the rate of change in pupil diameter. These metrics were calculated from the eye-tracker's raw data output. Gaze proportion was obtained by calculating the number of gaze points for a specific emotional stimulus as a percentage of the total number of gaze points; we calculated the gaze proportion in each 100 ms time bin (Mirman et al., 2008; Dink and Ferguson, 2015). The rate of change in pupil diameter was obtained by calculating the rate of change in pupil diameter in each 100 ms time bin compared to the baseline (Kret and Sjak-Shie, 2019; Reilly et al., 2019). The baseline value is the mean pupil diameter of each participant at 400 ms before the onset of picture viewing in each trial.

Eye-tracking data were cleaned to exclude data with a raw data loss rate overall or in individual trials exceeding 20%. Data cleaning thus excluded data for two participants and 23 trials. All analyses were performed using R Studio (2,022.02.1 + 461). The lmerTest (3.1–3) and lmer4 (4_1.1-29) R packages were used for mixed linear model analysis and GCA analysis. The version of the R language is 4.1.2 (2021-11-01). The hardware platform on which the above software runs is aarch64-apple-darwin20 (64-bit) running under macOS Monterey (12.3).

## Results

### Static analysis of gaze data

We first analyzed the gaze proportion of the HTA and LTA groups at different emotional stimuli using traditional static analysis methods, distinguished by different stimulus presentation duration. The different stimulus durations were obtained during the analysis stage by dividing the data for the 30 s presentation into multiple segments. The dependent variable is the average gaze proportion. Independent variables include trait anxiety (between-group variable: HTA, LTA), emotional stimulus type (within-group variable: neutral, positive, dysphoric, threat), and stimulus duration (within-group variable: 200, 400, 800, 1,600, 3,200, 6,400, 12,800, 25,600, 30,000 ms).

**Figure 2:**
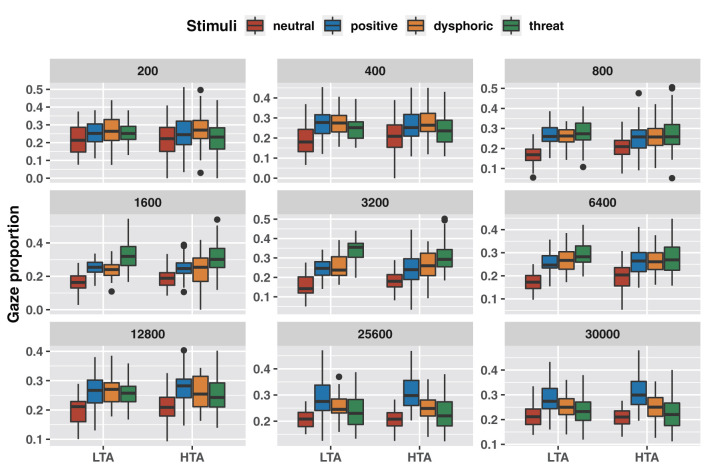
Differences in attentional bias between emotional stimuli with different stimulus durations.

Mixed analysis of variance results showed that trait anxiety did not affect the amount of attention to emotional stimuli across stimulus durations (Table 1). However, the results of pairwise comparisons corrected with the Bonferroni method showed that both groups invested less attention to neutral stimuli of more than 400 ms stimulus duration compared to positive and dysphoric stimuli; more attention to threat stimuli in the 1,600–6,400 ms stimulus duration; and more attention to positive stimuli in the 25,600–30,000 ms stimulus duration (Figure 2; Supplementary Table S2).

**Table 1:** Mixed analysis of variance results for trait anxiety (TA), stimulus, and duration.

**Effect**	***DFn***	***DFd***	***F***	***p***	**η^2^**
TA	1	47	0.225	0.637	0.000
Stimulus	3	141	30.567	<0.001	0.204
Duration	1.04	48.93	69.649	<0.001	0.000
TA:Stimulus	3	141	0.403	0.751	0.003
TA:Duration	1.04	48.93	0.159	0.702	0.000
Stimulus:Duration	6.68	313.99	9.104	<0.001	0.105
TA:Stimulus:Duration	6.68	313.99	0.652	0.705	0.008

These crude results showed partial changes in the characteristics of attentional bias over time, but did not reveal any effect of trait anxiety on attentional bias.

### Dynamic analysis of time-series data

**Figure 3:**
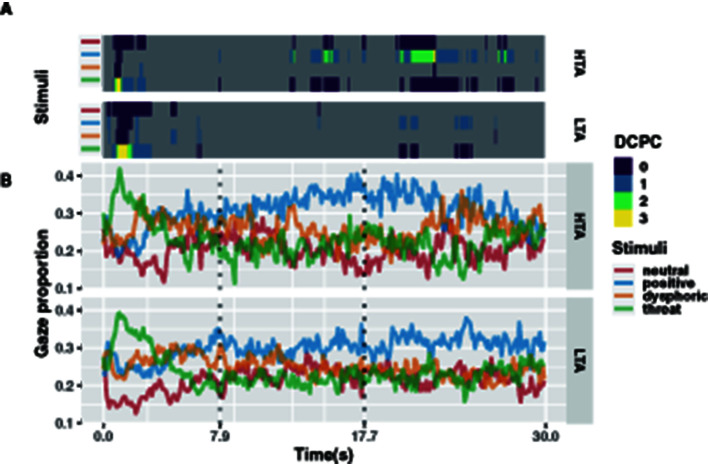
For each 100 ms bin prediction model, dominance count of pairwise comparisons (DCPC, adjusted *p-value* < 0.05) of HTA and LTA groups **(A)**. Gaze proportions of the HTA and LTA groups at each emotional stimulus in each 100 ms bin **(B)**.

For a more detailed dynamic analysis, we analyzed the gaze proportion for different emotional stimuli in 100 ms time bins (Figure 3B). For each time bin, we constructed a mixed linear model to examine the difference in gaze proportion between the HTA and LTA groups for different emotional stimuli at different time points. Fixed effects include the type of emotional stimulus and trait anxiety. Random effects include participant differences and item differences.

Corrected pairwise tests were used to verify specific differences in the gaze proportion across emotional stimuli. To further reduce the family-wise error, all results were further corrected by the Holm-Bonferroni method, and the alpha coefficient was set to 0.05 (Ludbrook, 1998). Since thousands of pairwise comparisons were performed, we performed a dominance count of pairwise comparisons (DCPC) to visualize the results. The DCPC shows the number of times an emotional stimulus was significantly greater in all pairwise comparisons in a given time bin (Figure 3A; Supplementary Table S3). Taking 2 s duration as the threshold, the results showed three distinct time periods in the gaze proportion patterns of the HTA and LTA groups for different emotional stimuli. During the 0–7.9 s period, both groups showed a very clear pattern of vigilance-avoidance attention to threatening stimuli, and the HTA group seemed to show a higher avoidance trend (fewer DCPC). During the 7.9–17.7 s period, the HTA group showed partial attention bias toward positive stimuli, whereas the LTA group show no attentional bias toward any particular emotional stimulus. Finally, during the 17.7–30 s period, both groups showed a greater preference for positive stimuli but this attentional bias was more pronounced in the HTA group.

### Growth curve analysis of gaze proportion

To further verify the above results, we performed a GCA, which provides a good fit to high-frequency non-linear continuous data and avoids the interpretation of complex multiple comparisons (Mirman et al., 2008; Curran et al., 2010; Mirman, 2017). This study's GCA analysis draws on the method of Mirman (2017) by using natural polynomials as time variables, thus allowing for fitting non-linear variations while preventing multicollinearity. Since the gaze proportion data are calculated based on a binary logistic value of whether the gaze point was hit or not, we performed an empirical log-transformation of gaze proportions for better analysis. Fixed effects in the GCA model include trait anxiety, emotional stimulus type, and a natural polynomial of time. Random effects include participants and items. Because the time-series is long (30 s), a good fit requires using a model of order 10 or higher, and such a complex model is difficult to interpret. Therefore, we divide the time into three periods based on results reported in the previous section. This allows us to a model of order four or below for the analysis, ensuring a good fit and reducing the difficulty of interpretation. For all three time periods, we performed model comparisons for optimal selection (Table 2). Finally, we used a fourth-order model for the 0–7.9 s period and a second-order model for the other two periods. The LTA group served as the baseline for estimating parameters for the HTA group (Table 3).

**Table 2:** Gaze proportion GCA model comparisons.

**Model**	**χ^2^**	***Df***	***P***
0–7.9 s period			
Model.0	1,419.08	4	<0.001
Model.1	1,314.18	8	<0.001
Model.2	141.54	8	<0.001
Model.3	403.52	8	<0.001
Model.4	145.20	8	<0.001
**7.9–17.7 s period**			
Model.0	2,559.46	4	<0.001
Model.1	176.10	8	<0.001
Model.2	17.34	8	0.027
**17.7–30.0 s period**			
Model.0	4,807.99	4	<0.001
Model.1	295.35	8	<0.001
Model.2	125.53	8	<0.001

*Model*.0:*Elog of gaze proportion* ~ *S* × *TA*+( 1 | *P*) .

*Model*.1:*Elog of gaze proportion* ~ *OT*_1_× *S* × *TA*+( 1 | *P*) .

*Model*.2:*Elog of gaze proportion* ~ (*OT*_1_+*OT*_2_) × *S* × *TA*+( 1 | *P*) .

*Model*.3:*Elog of gaze proportion* ~ (*OT*_1_+*OT*_2_+*OT*_3_) × *S* × *TA*+( 1 | *P*) .

*Model*.4:*Elog of gaze proportion* ~ (*OT*_1_+*OT*_2_+*OT*_3_+*OT*_4_) × *S* × *TA*+( 1 | *P*).

Stimulus (S), trait anxiety (TA), natural polynomials (OT), participant differences (P).

**Table 3:** Parameter estimation of gaze proportion GCA.

**Interaction effect**	**Estimate**	***SE***	***t***	***p***
**0–7.9 s period**				
**Threat**				
Intercept	−0.05	0.03	−1.65	0.100
Linear	1.46	0.25	5.81	<0.001
Quadratic	1.04	0.25	4.15	<0.001
Cubic	−1.02	0.25	−4.08	<0.001
Quartic	−0.62	0.25	−2.46	0.014
**Dysphoric**				
Intercept	−0.09	0.03	−2.85	0.005
Linear	−1.75	0.25	−6.97	<0.001
Quadratic	−0.15	0.25	−0.61	0.541
Cubic	0.71	0.25	2.84	0.005
Quartic	−0.32	0.25	−1.29	0.199
**Positive**				
Intercept	0.08	0.03	2.50	0.013
Linear	0.14	0.25	0.57	0.568
Quadratic	−1.00	0.25	−3.97	<0.001
Cubic	−0.07	0.25	−0.26	0.793
Quartic	1.11	0.25	4.41	<0.001
**Neutral**				
Intercept	0.14	0.03	4.55	<0.001
Linear	−0.09	0.25	−0.35	0.730
Quadratic	0.23	0.25	0.91	0.361
Cubic	0.27	0.25	1.08	0.279
Quartic	0.00	0.25	0.00	0.998
**7.9–17.7 s period**				
**Threat**				
Intercept	−0.04	0.03	−1.41	0.158
Linear	0.03	0.25	0.12	0.909
Quadratic	0.44	0.25	1.75	0.080
**Dysphoric**				
Intercept	−0.06	0.03	−1.96	0.052
Linear	0.31	0.25	1.21	0.225
Quadratic	−0.50	0.25	−1.98	0.048
**Positive**				
Intercept	0.24	0.03	7.71	<0.001
Linear	0.43	0.25	1.73	0.085
Quadratic	−0.22	0.25	−0.88	0.378
**Neutral**				
Intercept	0.02	0.03	0.80	0.428
Linear	−0.90	0.25	−3.58	<0.001
Quadratic	−0.22	0.25	−0.86	0.387
**17.7–30.0 s period**				
**Threat**				
Intercept	−0.07	0.03	−2.35	0.020
Linear	−1.98	0.25	−7.90	<0.001
Quadratic	0.27	0.25	1.07	0.286
**Dysphoric**				
Intercept	0.17	0.03	5.74	<0.001
Linear	1.26	0.25	5.03	<0.001
Quadratic	−0.64	0.25	−2.57	0.010
**Positive**				
Intercept	0.14	0.03	4.68	<0.001
Linear	−0.84	0.25	−3.34	0.001
Quadratic	0.17	0.25	0.69	0.492
**Neutral**				
Intercept	−0.15	0.03	−5.08	<0.001
Linear	1.51	0.25	6.03	<0.001
Quadratic	0.37	0.25	1.46	0.144

**Figure 4:**
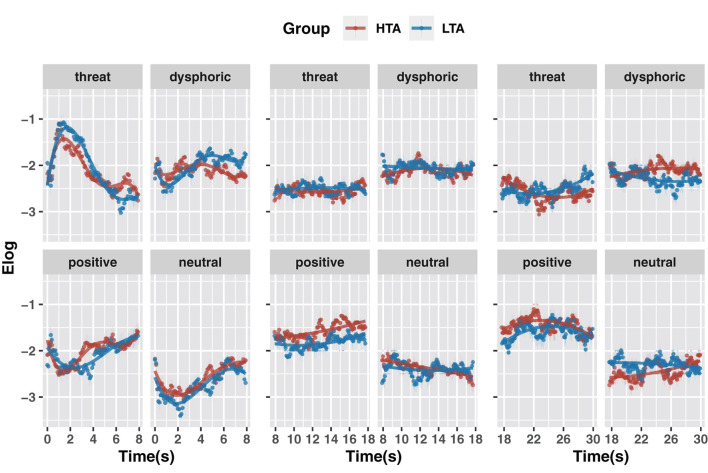
GCA model fits of the gaze data on emotional stimuli for the HTA and LTA groups in 0–7.9 s period **(A)**, 7.9–17.7 s period **(B)** and 17.7–30.0 s period **(C)**.

For the 0–7.9 s period, we used a fourth-order polynomial to construct the model. As shown in Figure 4A, for positive stimuli the HTA (vs. LTA) group showed more attention (intercept term: β = 0.08, *t* = 2.50, *p* = 0.013), a lower rate of rise (quadratic term interaction: β = −1.00, *t* = −3.97, *p* < 0.001) and more significant end-of-period growth trend (quartic term interaction: β = 1.11, *t* = 4.41, *p* < 0.001). For dysphoric stimuli the HTA group showed less attention (intercept term: β = −0.09, *t* = −2.85, *p* = 0.005) and less positive growth (linear term interaction: β = −1.75, *t* = −6.97, *p* < 0.001). For threatening stimuli the HTA group had fewer decreasing trends (linear term interaction: β = 1.46, *t* = 5.81, *p* < 0.001), a lower rate of decline (quadratic term interaction: β = 1.04, *t* = 4.15, *p* < 0.001), and more significant end-of-period decline trend (cubic term interaction: β = −1.02, *t* = −4.08, *p* < 0.001; quartic term interaction: β = −0.62, *t* = −2.46, *p* < 0.014). Finally, for neutral stimuli the HTA group showed more attention (intercept term: β = 0.14, *t* = 4.55, *p* < 0.001). These results suggest that trait anxiety affected attention to emotional stimuli during the 0–7.9 s time window. Specifically, compared to the LTA group, the HTA group gave less attention to dysphoric stimuli and more attention to positive and neutral stimuli. At the end of the period (after 7 s), the HTA group showed significant avoidance of threatening stimuli and an attentional bias toward positive stimuli.

For the 7.9–17.7 s period, we constructed models using second-order polynomials (Figure 4B). Compared to the LTA group, the HTA group showed more attention to positive stimuli (intercept term: β = 0.24, *t* = 7.71, *p* < 0.001). For neutral stimuli, the HTA group showed a more negative growth (linear term interaction: β = −0.90, *t* = −3.58, *p* < 0.001). These results suggest that within the 7.9–17.7 s time window, trait anxiety is associated with a rapid rise in attention to positive stimuli and a mild decline in attention to neutral stimuli.

For the 17.7–30.0 s period, we constructed models using second-order polynomials (Figure 4C). Compared to the LTA group, for positive stimuli the HTA group showed more attention (intercept term: β = 0.14, *t* = 4.68, *p* < 0.001) and a lower growth trend (linear term interaction: β = −0.84, *t* = −3.34, *p* = 0.001); for dysphoric stimuli, the HTA group showed more attention (intercept term: β = 0.17, *t* = 5.74, *p* < 0.001), a higher growth trend (linear term interaction: β = 1.26, *t* = 5.03, *p* < 0.001) and a higher rate of decline (quadratic term interaction: β = −0.64, *t* = −2.57, *p* = 0.010); for threat stimuli, the HTA group showed less attention (intercept term: β = −0.07, *t* = −2.35, *p* = 0.020) and a lower growth trend (linear term interaction: β = −1.98, *t* = −7.90, *p* < 0.001); and for neutral stimuli the HTA group showed less attention (intercept term: β = −0.15, *t* = −5.08, *p* < 0.001) and a higher growth trend (linear term interaction: β = 1.51, *t* = 6.03, *p* < 0.001). These results suggest that during the 17.7–30.0 s time window, the HTA group gave more attention to positive and dysphoric stimuli and less attention to threatening and neutral stimuli.

### Growth curve analysis of pupil diameter

**Figure 5:**
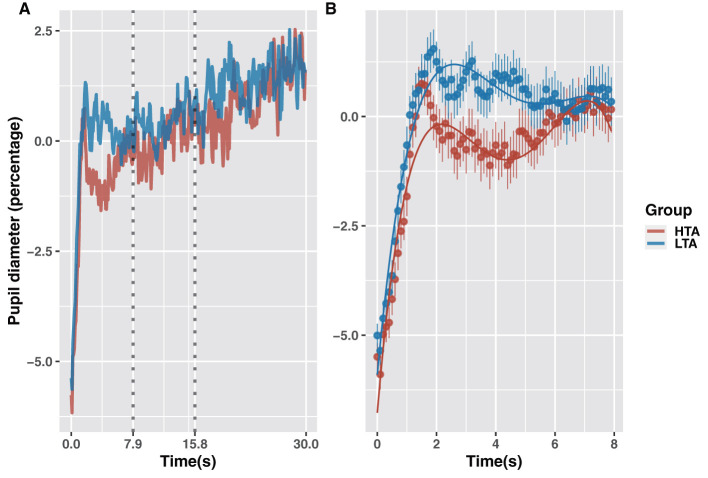
Rate of change in pupil diameter in the HTA and LTA groups **(A)**. GCA model fits for pupil-diameter data of the HTA and LTA groups (0–7.9 s) **(B)**.

To calculate time-series data on the pupil diameter change rate during the 30 s of stimulus presentation, we used pupil diameter at 400 ms before stimulus onset as the baseline (Figure 5A). As in the analyses reported above, the time-series data were divided into three periods, and for each period, the optimal model was selected by comparing from the 0 model to the fourth-order model (Table 4). Fixed effects in the GCA model include trait anxiety, emotional stimulus type, and a natural polynomial of time. Random effects include participants and items. The LTA group was used as the baseline for estimating parameters for the HTA group (Table 5).

**Table 4:** Pupil diameter GCA model comparisons.

**Model**	**χ^2^**	***Df***	***p***
**0–7.9 s period**			
Model.0	759.45	1	<0.001
Model.1	–	–	–
Model.2	–	–	–
Model.3	389.80	2	<0.001
Model.4	163.98	2	<0.001
7.9–17.7s periodz			
Model.0	6.59	1	0.010
Model.1	–	–	–
Model.2	–	–	–
**17.7–30.0s period**			
Model.0	–	–	–
Model.1	–	–	–
Model.2	24.92	1	<0.001

*Model*.0:*Pupil diameter* ~ *S* × *TA*+( 1 | *P*) .

*Model*.1:*Pupil diameter* ~ *OT*_1_× *S* × *TA*+( 1 | *P*) .

*Model*.2:*Pupil diameter* ~ (*OT*_1_+*OT*_2_) × *S* × *TA*+( 1 | *P*) .

*Model*.3:*Pupil diameter* ~ (*OT*_1_+*OT*_2_+*OT*_3_) × *S* × *TA*+( 1 | *P*) .

*Model*.4:*Pupil diameter* ~ (*OT*_1_+*OT*_2_+*OT*_3_+*OT*_4_) × *S* × *TA*+( 1 | *P*).

Stimulus (S), trait anxiety (TA), natural polynomials (OT), participant differences (P).

**Table 5:** Parameter estimation of pupil diameter GCA.

**Fixed effect**	**Estimate**	***SE***	***t***	***p***
**0–7.9 s period**				
Intercept	−0.009	0.007	−1.41	0.161
Linear	0.020	0.007	2.97	<0.001
Quadratic	0.034	0.007	5.16	<0.001
Cubic	−0.008	0.007	−1.22	0.222
Quartic	−0.020	0.007	−3.03	0.002
**7.9–17.7 s period**				
Intercept	−0.003	0.007	−0.43	0.672
Linear	<0.001	0.007	0.01	0.996
Quadratic	−0.010	0.007	−1.64	0.102
**17.7–30.0 s period**				
Intercept	−0.002	0.007	−0.38	0.703
Linear	0.039	0.008	5.00	<0.001
Quadratic	<0.001	0.008	0.03	0.973

GCA analysis revealed that trait anxiety caused a significant difference in pupil diameter between 0 and 7.9 s (Figure 5B). Compared to the LTA group, the HTA group showed no difference in mean pupil diameter (intercept term: β = −0.01, *t* = −1.41, *p* = 0.161), a higher growth trend (linear term: β = 0.02, *t* = 2.97, *p* = 0.003), and a higher rate of growth (quadratic term interaction: β = 0.03, *t* = 5.16, *p* < 0.001). Combined with the findings shown in Figure 4B, these results indicate that the HTA group demonstrated significant fluctuations in pupil diameter (falling then rising) during the 2–6 s period. This time window corresponds to the avoidance stage of attention to threatening stimuli.

## Discussion

This study explores the time course of attentional selection of emotional stimuli in high and low trait anxiety groups. It reveals significant differences in the trends of attention to particular emotional stimuli with time and between the two groups. During the 0–7.9 s period, both groups showed similar attention patterns: a rapid increase followed by a decrease in attention to threatening stimuli (peaking in the 1–2 s period); a rapid decrease followed by an increase in attention to neutral stimuli (reaching its minimum in the 1–2 s period); and initially moderate then a slow increase in attention to positive and dysphoric stimuli. The HTA group showed more attention to positive stimuli over the full presentation duration (30 s) and more attention to dysphoric stimuli during the 17.7–30.0 s period. For threatening stimuli, the HTA showed a significant vigilance-avoidance pattern during 0–7.9 s and further avoidance after 7 s, with a rapid shift in attention toward positive stimuli. Relatedly, compared to the LTA group the HTA group showed a more significant decrease in pupil diameter during the declining stage of attention to threatening stimuli (2–6 s period).

### The effect of temporal factors on attentional bias

This study further investigates the temporal effect using more detailed temporal information. Bin-by-bin analysis of the gaze proportion and GCA analysis further supports the idea of a significant temporal effect on attentional selection of emotional stimuli (Kellough et al., 2008). Specifically, attention to threatening stimuli peaked rapidly at around 2 s after stimulus presentation, then dropped back to an average level at around 6 s. Displaying the opposite pattern, attention to neutral stimuli quickly reaching its minimum at around 2 s, then rebounded to an average level at around 6 s. Attention to positive stimuli gradually rose to become dominant after 10 s.

Many previous studies have found no effect of trait anxiety on selective attention (Berggren et al., 2012; Quigley et al., 2012; Nelson et al., 2015). However, researchers have shown that manipulating the duration of stimulus presentation can reveal the effect of trait anxiety on attention (Mogg et al., 2004; Koster et al., 2005; Sagliano et al., 2014). This study further corroborates the significant effect of trait anxiety on attentional selection through a time-course design. The results indicate a time-varying effect of trait anxiety on attention to positive, dysphoric, and threatening emotional stimuli during the 30 s period, with differences particularly manifesting during the 7.9–30.0 s period.

These results suggest that the time factor cannot be ignored. There is evidently a temporal effect on the differences in attentional selection patterns for emotional stimuli between HTA and LTA individuals. Such differences are difficult to detect when the temporal factor is ignored, which explains why some studies have found no effect of trait anxiety on attention to emotional stimuli.

### Attentional bias to threat

Koster et al. (2006) showed that trait anxiety affects the engagement and disengagement of attentional bias for moderately and highly threatening pictures in an exogenous cueing task. Specifically, HTA individuals showed stronger attentional engagement and weaker attentional disengagement in response to threatening stimuli in the early stages (duration = 100 ms) but a stronger tendency to attentional avoidance of threat in the later stages (duration = 200, 500 ms). However, numerous studies have shown that trait anxiety does not affect attention to threatening stimuli in longer stimulus presentation conditions (Berggren et al., 2012; Quigley et al., 2012; Nelson et al., 2015). This study further detailed how trait anxiety affects attention to threatening stimuli through a time-series analysis of a free-viewing task. The HTA group showed the vigilance-avoidance attention pattern for threatening stimuli in the early period (0–7.9 s) and a more pronounced tendency to avoid compared to the LTA group after 7 s.

Notably, pupil-diameter analysis revealed a significant decrease and rebound in the HTA group during the 2–8 s period, corresponding to the stage when attention to threatening stimuli dropped from its highest to lowest level. Studies have shown that pupil diameter is closely related to neural activity (Murphy et al., 2014; Joshi et al., 2016). For instance, the degree of emotional arousal prompted by a stimulus is reflected by change in the pupil diameter (Bradley et al., 2008; Van Steenbergen et al., 2011). Therefore, results from the pupil-diameter analysis further suggest that trait anxiety affects attention to threatening stimuli. In terms of attentional bias, HTA induced more significant vigilance-avoidance. In other respects, it also triggered greater changes in neural activity in response to emotional arousal.

### Attentional bias to positive and dysphoric

Mansell et al. (2002) found that HTA was associated with selective attention to dysphoric rather than positive social-evaluative words. Relatedly, Veerapa et al. (2020) found that attentional maintenance bias for dysphoric pictures increases with trait anxiety. Supporting these earlier findings, HTA was associated in this study with more attention to dysphoric stimuli compared to the LTA group during 17.7–30.0 s. Surprisingly, we found that the HTA group showed more attention bias to positive stimuli compared to the LTA group throughout the 30 s stimulus presentation period. This bias appeared rapidly after 7 s, then showed a continuously increasing trend during 7.9–17.7 s. Such results have rarely been reported in previous studies. A possible explanation is that most prior research uses competing-attention tasks whereas we used a free-viewing task. Moreover, the rapid emergence of attentional bias toward positive stimuli after 7 s was not observed in previous studies that used much shorter stimulus presentations.

Trait anxiety seems to be associated with greater attentional bias toward dysphoric stimuli when the presentation period is shorter (MacLeod et al., 2002; Mathews and MacLeod, 2005; Haddadi and Besharat, 2010). However, this study found that trait anxiety induced a greater attentional bias toward positive stimuli in a longer presentation period. Considering that the timing of this bias' emergence overlaps with the timing of threat-stimulus avoidance, this bias may be closely related to attentional avoidance of threat stimuli. Essentially, attentional bias toward positive stimuli may be an effective strategy for avoiding attention to threatening stimuli. Another explanation is that the vulnerability characterizing trait anxiety amplifies attention to not only negative class stimuli but also positive class stimuli. In other words, trait anxiety shows an emotional susceptibility to positive and negative stimuli.

## Conclusion

This study aimed to better understand the effects of trait anxiety on attention to emotional stimuli. Evidence from the present study suggests that there is a significant temporal effect of trait anxiety on attention to emotional stimuli and that this effect is mainly present in the period after 7 s. Specifically, high trait anxiety showed a more significant tendency to avoid threatening stimuli in the early period; high trait anxiety showed more changes in neural activity in response to attentional avoidance of threatening stimuli; high trait anxiety showed a significant attentional bias toward positive stimuli in the middle and late periods.

These findings provide important insights into the role of attention bias in trait anxiety. The strength of the study is the in-depth analysis of the temporal effect of trait anxiety on attention to emotional stimuli. A limitation of this study is that only a single scale was used to measure the level of trait anxiety. Potential future research avenues include further exploration of the temporal effects of trait anxiety on the attentional characteristics of threatening stimuli, as well as analysis of the possible causes of such temporal effects by designing operationalized variable contrasts; to examine the relationship between trait anxiety and attention to positive stimuli and to further verify whether attention to positive stimuli is a strategy for avoiding threatening stimuli.

## Data availability statement

The datasets presented in this article are not readily available because of privacy protection for the participants. Requests to access the datasets should be directed to the corresponding author.

## Ethics statement

The studies involving human participants were reviewed and approved by Medical Ethics Committee of Xijing Hospital, Fourth Military Medical University. The patients/participants provided their written informed consent to participate in this study. Written informed consent was obtained from the individual(s) for the publication of any potentially identifiable images or data included in this article.

## Author contributions

CX and DM: conceptualization. CX and YZ: methodology. CX and HL: formal analysis and investigation. YZ and HL: data collection and collation. CX: writing—original draft preparation. DM and XZ: writing—review and editing. DM: funding acquisition. XZ: supervision. All authors contributed to the article and approved the submitted version.

## Funding

This work was supported by grants A study of brain mechanisms in the assessment and training of emotional stability from Fourth Military Medical University (AWS17J012MDM).

## Conflict of interest

The authors declare that the research was conducted in the absence of any commercial or financial relationships that could be construed as a potential conflict of interest.

## Publisher's note

All claims expressed in this article are solely those of the authors and do not necessarily represent those of their affiliated organizations, or those of the publisher, the editors and the reviewers. Any product that may be evaluated in this article, or claim that may be made by its manufacturer, is not guaranteed or endorsed by the publisher.
