# Supplementary material for: Trait anxiety affects attentional bias to emotional stimuli across time: A growth curve analysis
Source: Front Neurosci. 2022 Sep 14;16:972892. doi: 10.3389/fnins.2022.972892 (PMC9516103; doi:10.3389/fnins.2022.972892)
Supplement: Supplementary file 1 [file Table_1.DOCX]

**Supplementary Table S1. Picture Stimulation List**

| **Picture Name** | **IAPS Number** | **Emotional Valence (Mean)** | **Emotional Valence (SD)** | **Emotional arousal level (Mean)** | **Emotional arousal level (SD)** |
| --- | --- | --- | --- | --- | --- |
| Snake | 1120 | 3.79 | 1.93 | 6.93 | 1.68 |
| PitBull | 1300 | 3.55 | 1.78 | 6.79 | 1.84 |
| Women | 1340 | 7.13 | 1.57 | 4.75 | 2.31 |
| NeuWoman | 2038 | 5.09 | 1.35 | 2.94 | 1.93 |
| Girls | 2091 | 7.68 | 1.43 | 4.51 | 2.28 |
| NeuMan | 2102 | 5.16 | 0.96 | 3.03 | 1.87 |
| GrievingFem | 2141 | 2.44 | 1.64 | 5 | 2.03 |
| Father | 2165 | 7.63 | 1.48 | 4.55 | 2.55 |
| Hospital | 2205 | 1.95 | 1.58 | 4.53 | 2.23 |
| Bride | 2208 | 7.35 | 1.68 | 5.68 | 2.34 |
| Boys | 2224 | 7.24 | 1.58 | 4.85 | 2.11 |
| Girl | 2276 | 2.67 | 1.66 | 4.63 | 1.93 |
| Family | 2299 | 7.27 | 1.53 | 3.95 | 2.22 |
| Father | 2339 | 6.72 | 1.34 | 4.16 | 1.9 |
| Family | 2340 | 8.03 | 1.26 | 4.9 | 2.2 |
| Factoryworker | 2393 | 4.87 | 1.06 | 2.93 | 1.88 |
| Men | 2397 | 4.98 | 1.11 | 2.77 | 1.74 |
| SadGirls | 2455 | 2.96 | 1.79 | 4.46 | 2.12 |
| Couple | 2501 | 6.89 | 1.78 | 3.09 | 2.21 |
| Woman | 2700 | 3.19 | 1.56 | 4.77 | 1.97 |
| SadChildren | 2703 | 1.91 | 1.26 | 5.78 | 2.25 |
| Shopping | 2745 | 5.31 | 1.08 | 3.26 | 1.96 |
| Funeral | 2799 | 2.42 | 1.41 | 5.02 | 1.99 |
| Gun | 2811 | 2.17 | 1.38 | 6.9 | 2.22 |
| Tourist | 2850 | 5.22 | 1.39 | 3 | 1.94 |
| CryingBoy | 2900 | 2.45 | 1.42 | 5.09 | 2.15 |
| DyingMan | 3230 | 2.02 | 1.3 | 5.41 | 2.21 |
| Attack | 3500 | 2.21 | 1.34 | 6.99 | 2.19 |
| Romance | 4599 | 7.12 | 1.48 | 5.69 | 1.94 |
| Couple | 4700 | 6.91 | 1.94 | 4.05 | 1.9 |
| Mushroom | 5500 | 5.42 | 1.58 | 3 | 2.42 |
| Flowers | 5731 | 5.39 | 1.58 | 2.74 | 1.95 |
| AimedGun | 6260 | 2.44 | 1.54 | 6.93 | 1.93 |
| Abduction | 6312 | 2.48 | 1.52 | 6.37 | 2.3 |
| Attack | 6313 | 1.98 | 1.38 | 6.94 | 2.23 |
| Attack | 6350 | 1.9 | 1.29 | 7.29 | 1.87 |
| Attack | 6510 | 2.46 | 1.58 | 6.96 | 2.09 |
| Attack | 6560 | 2.16 | 1.41 | 6.53 | 2.42 |
| Attack | 6562 | 3.19 | 1.34 | 5.08 | 2.39 |
| Gang | 6821 | 2.38 | 1.72 | 6.29 | 2.02 |
| Mug | 7009 | 4.93 | 1 | 3.01 | 1.97 |
| Baskets | 7041 | 4.99 | 1.12 | 2.6 | 1.78 |
| Fork | 7080 | 5.27 | 1.09 | 2.32 | 1.84 |
| AbstractArt | 7185 | 4.97 | 0.87 | 2.64 | 2.04 |
| HappyTeens | 8461 | 7.22 | 1.53 | 4.69 | 2.2 |
| Cemetery | 9220 | 2.06 | 1.54 | 4 | 2.09 |
| Soldier | 9421 | 2.21 | 1.45 | 5.04 | 2.15 |
| Boys | 9530 | 2.93 | 1.84 | 5.2 | 2.26 |
