# Supplementary material for: Trait anxiety affects attentional bias to emotional stimuli across time: A growth curve analysis
Source: Front Neurosci. 2022 Sep 14;16:972892. doi: 10.3389/fnins.2022.972892 (PMC9516103; doi:10.3389/fnins.2022.972892)
Supplement: Supplementary file 2 [file Table_2.DOCX]

**Supplementary Table S2. Pairwise comparisons of Gaze proportion between stimuli at different stimulus presentation times (Bonferroni method)**

| Duration (ms) | Lower Group | Higher Group | n1 | n2 | p.adj |
| --- | --- | --- | --- | --- | --- |
| 200 | neutral | positive | 86 | 83 | 0.040 |
| 200 | neutral | dysphoric | 86 | 83 | 0.001 |
| 400 | neutral | positive | 85 | 82 | <0.001 |
| 400 | neutral | dysphoric | 85 | 81 | <0.001 |
| 400 | neutral | threat | 85 | 81 | <0.001 |
| 800 | neutral | positive | 78 | 81 | <0.001 |
| 800 | neutral | dysphoric | 78 | 83 | <0.001 |
| 800 | neutral | threat | 78 | 82 | <0.001 |
| 1600 | neutral | positive | 82 | 79 | <0.001 |
| 1600 | neutral | dysphoric | 82 | 85 | <0.001 |
| 1600 | neutral | threat | 82 | 84 | <0.001 |
| 1600 | positive | threat | 79 | 84 | <0.001 |
| 1600 | dysphoric | threat | 85 | 84 | <0.001 |
| 3200 | neutral | positive | 84 | 86 | <0.001 |
| 3200 | neutral | dysphoric | 84 | 86 | <0.001 |
| 3200 | neutral | threat | 84 | 83 | <0.001 |
| 3200 | positive | threat | 86 | 83 | <0.001 |
| 3200 | dysphoric | threat | 86 | 83 | <0.001 |
| 6400 | neutral | positive | 84 | 81 | <0.001 |
| 6400 | neutral | dysphoric | 84 | 86 | <0.001 |
| 6400 | neutral | threat | 84 | 85 | <0.001 |
| 6400 | positive | threat | 81 | 85 | 0.024 |
| 12800 | neutral | positive | 86 | 83 | <0.001 |
| 12800 | neutral | dysphoric | 86 | 85 | <0.001 |
| 12800 | neutral | threat | 86 | 86 | <0.001 |
| 25600 | neutral | positive | 81 | 86 | <0.001 |
| 25600 | neutral | dysphoric | 81 | 84 | <0.001 |
| 25600 | positive | dysphoric | 86 | 84 | <0.001 |
| 25600 | neutral | threat | 81 | 86 | 0.022 |
| 25600 | positive | threat | 86 | 86 | <0.001 |
| 30000 | neutral | positive | 82 | 85 | <0.001 |
| 30000 | neutral | dysphoric | 82 | 85 | <0.001 |
| 30000 | positive | dysphoric | 85 | 85 | <0.001 |
| 30000 | positive | threat | 85 | 85 | <0.001 |
