# Supplementary material for: Trait anxiety affects attentional bias to emotional stimuli across time: A growth curve analysis
Source: Front Neurosci. 2022 Sep 14;16:972892. doi: 10.3389/fnins.2022.972892 (PMC9516103; doi:10.3389/fnins.2022.972892)
Supplement: Supplementary file 3 [file Table_3.DOCX]

**Supplementary Table S3 Pairwise comparisons of Gaze proportion between stimuli at each time-bin of the HTA and LTA groups (Holm-Bonferroni method)**

| **Contrast** | **Trait anxiety group** | **Estimate** | **SE** | **df** | **t.ratio** | **p.value** | **Time** |
| --- | --- | --- | --- | --- | --- | --- | --- |
| neutral - positive | LTA | -0.1588338 | 0.02820704 | 3612 | -5.630998 | 1.22E-07 | 300 |
| neutral - positive | LTA | -0.1490047 | 0.02816749 | 3616 | -5.2899535 | 7.81E-07 | 400 |
| neutral - dysphoric | LTA | -0.1340746 | 0.02816749 | 3616 | -4.759905 | 1.20E-05 | 400 |
| neutral - threat | LTA | -0.1271228 | 0.02829203 | 3616 | -4.4932359 | 4.28E-05 | 600 |
| neutral - threat | LTA | -0.15197 | 0.02831912 | 3608 | -5.3663395 | 5.17E-07 | 700 |
| neutral - threat | HTA | -0.1577609 | 0.027577 | 3608 | -5.7207403 | 7.48E-08 | 700 |
| neutral - threat | LTA | -0.1790468 | 0.02824859 | 3608 | -6.3382568 | 7.63E-09 | 800 |
| neutral - threat | HTA | -0.1748199 | 0.02738677 | 3608 | -6.38337 | 7.23E-09 | 800 |
| dysphoric - threat | HTA | -0.1258893 | 0.02738677 | 3608 | -4.596718 | 2.63E-05 | 800 |
| neutral - threat | LTA | -0.2053936 | 0.02814203 | 3616 | -7.2984624 | 6.35E-09 | 900 |
| dysphoric - threat | LTA | -0.1405158 | 0.02814203 | 3616 | -4.9930922 | 3.72E-06 | 900 |
| neutral - threat | HTA | -0.1815951 | 0.02728534 | 3616 | -6.6554086 | 6.54E-09 | 900 |
| positive - threat | HTA | -0.1366122 | 0.02728534 | 3616 | -5.0067997 | 3.47E-06 | 900 |
| dysphoric - threat | HTA | -0.1258885 | 0.02728534 | 3616 | -4.6137792 | 2.43E-05 | 900 |
| neutral - threat | LTA | -0.2088128 | 0.02826795 | 3592 | -7.3869115 | 5.51E-09 | 1000 |
| dysphoric - threat | LTA | -0.1643732 | 0.02826795 | 3592 | -5.814827 | 4.51E-08 | 1000 |
| positive - threat | LTA | -0.1408182 | 0.02826795 | 3592 | -4.9815516 | 3.94E-06 | 1000 |
| neutral - threat | HTA | -0.2026578 | 0.02734088 | 3592 | -7.4122626 | 5.51E-09 | 1000 |
| positive - threat | HTA | -0.147681 | 0.02734088 | 3592 | -5.4014739 | 4.27E-07 | 1000 |
| dysphoric - threat | HTA | -0.1266147 | 0.02734088 | 3592 | -4.6309661 | 2.24E-05 | 1000 |
| neutral - threat | LTA | -0.2046332 | 0.02824801 | 3588 | -7.2441622 | 5.38E-09 | 1100 |
| dysphoric - threat | LTA | -0.1533369 | 0.02824801 | 3588 | -5.4282356 | 3.69E-07 | 1100 |
| positive - threat | LTA | -0.1490505 | 0.02824801 | 3588 | -5.2764959 | 8.39E-07 | 1100 |
| neutral - threat | HTA | -0.2133461 | 0.02735102 | 3588 | -7.8002971 | 5.37E-09 | 1100 |
| positive - threat | HTA | -0.1471016 | 0.02735102 | 3588 | -5.3782853 | 4.84E-07 | 1100 |
| dysphoric - threat | HTA | -0.1375364 | 0.02735102 | 3588 | -5.028567 | 3.10E-06 | 1100 |
| neutral - threat | LTA | -0.2190862 | 0.02815721 | 3620 | -7.7808194 | 6.49E-09 | 1200 |
| dysphoric - threat | LTA | -0.1510736 | 0.02815721 | 3620 | -5.3653607 | 5.20E-07 | 1200 |
| positive - threat | LTA | -0.1503053 | 0.02815721 | 3620 | -5.3380754 | 6.03E-07 | 1200 |
| neutral - threat | HTA | -0.1989641 | 0.02721074 | 3620 | -7.3119689 | 6.49E-09 | 1200 |
| positive - threat | HTA | -0.138743 | 0.02721074 | 3620 | -5.0988349 | 2.15E-06 | 1200 |
| neutral - threat | LTA | -0.216693 | 0.02833877 | 3580 | -7.6465219 | 5.11E-09 | 1300 |
| dysphoric - threat | LTA | -0.1492305 | 0.02833877 | 3580 | -5.2659496 | 8.88E-07 | 1300 |
| positive - threat | LTA | -0.1416445 | 0.02833877 | 3580 | -4.9982584 | 3.62E-06 | 1300 |
| neutral - threat | HTA | -0.1619532 | 0.02719298 | 3580 | -5.9556991 | 2.21E-08 | 1300 |
| neutral - threat | LTA | -0.2164621 | 0.02841557 | 3596 | -7.6177266 | 5.65E-09 | 1400 |
| dysphoric - threat | LTA | -0.1650511 | 0.02841557 | 3596 | -5.8084721 | 4.67E-08 | 1400 |
| positive - threat | LTA | -0.1324809 | 0.02841557 | 3596 | -4.6622628 | 1.92E-05 | 1400 |
| neutral - threat | HTA | -0.1465788 | 0.0273932 | 3596 | -5.3509181 | 5.62E-07 | 1400 |
| neutral - threat | LTA | -0.2130138 | 0.02830668 | 3596 | -7.5252135 | 5.65E-09 | 1500 |
| dysphoric - threat | LTA | -0.1576416 | 0.02830668 | 3596 | -5.5690595 | 1.70E-07 | 1500 |
| positive - threat | LTA | -0.150471 | 0.02830668 | 3596 | -5.3157427 | 6.80E-07 | 1500 |
| neutral - threat | HTA | -0.1335758 | 0.02728822 | 3596 | -4.8949973 | 6.12E-06 | 1500 |
| neutral - threat | LTA | -0.2082151 | 0.02841134 | 3608 | -7.3285928 | 6.06E-09 | 1600 |
| positive - threat | LTA | -0.1369035 | 0.02841134 | 3608 | -4.8186205 | 8.96E-06 | 1600 |
| dysphoric - threat | LTA | -0.1288523 | 0.02841134 | 3608 | -4.5352437 | 3.52E-05 | 1600 |
| neutral - threat | HTA | -0.1382748 | 0.02742281 | 3608 | -5.0423287 | 2.89E-06 | 1600 |
| neutral - threat | LTA | -0.2193761 | 0.0283772 | 3600 | -7.7307163 | 5.79E-09 | 1700 |
| positive - threat | LTA | -0.1434958 | 0.0283772 | 3600 | -5.0567275 | 2.68E-06 | 1700 |
| neutral - threat | HTA | -0.1387647 | 0.02750957 | 3600 | -5.0442318 | 2.86E-06 | 1700 |
| neutral - threat | LTA | -0.2098391 | 0.02840693 | 3596 | -7.3868994 | 5.65E-09 | 1800 |
| positive - threat | LTA | -0.1286238 | 0.02840693 | 3596 | -4.5279022 | 3.64E-05 | 1800 |
| neutral - threat | HTA | -0.1473449 | 0.02750687 | 3596 | -5.3566607 | 5.45E-07 | 1800 |
| neutral - threat | LTA | -0.2176718 | 0.0283532 | 3604 | -7.6771498 | 5.92E-09 | 1900 |
| positive - threat | LTA | -0.1311397 | 0.0283532 | 3604 | -4.625216 | 2.30E-05 | 1900 |
| neutral - threat | HTA | -0.1458343 | 0.02739599 | 3604 | -5.3232003 | 6.53E-07 | 1900 |
| neutral - threat | LTA | -0.2240387 | 0.0282525 | 3628 | -7.9298704 | 6.78E-09 | 2000 |
| neutral - threat | HTA | -0.1386435 | 0.02730487 | 3628 | -5.0776101 | 2.40E-06 | 2000 |
| neutral - threat | LTA | -0.2208977 | 0.0283401 | 3584 | -7.7945285 | 5.24E-09 | 2100 |
| neutral - threat | LTA | -0.2361968 | 0.02824802 | 3596 | -8.3615353 | 5.65E-09 | 2200 |
| neutral - dysphoric | LTA | -0.14411 | 0.02824802 | 3596 | -5.1015962 | 2.12E-06 | 2200 |
| neutral - positive | LTA | -0.1325689 | 0.02824802 | 3596 | -4.6930325 | 1.66E-05 | 2200 |
| neutral - threat | LTA | -0.2190194 | 0.02875622 | 309.440287 | -7.6164198 | 3.05E-12 | 2300 |
| neutral - dysphoric | LTA | -0.150063 | 0.02875622 | 309.440287 | -5.2184529 | 1.98E-06 | 2300 |
| neutral - positive | LTA | -0.1327419 | 0.02875622 | 309.440287 | -4.6161123 | 3.39E-05 | 2300 |
| neutral - threat | HTA | -0.1339363 | 0.02780641 | 317.344704 | -4.8167409 | 1.34E-05 | 2300 |
| neutral - threat | LTA | -0.1980766 | 0.02833091 | 3608 | -6.991537 | 6.08E-09 | 2400 |
| neutral - threat | HTA | -0.1465999 | 0.02740582 | 3608 | -5.3492256 | 5.67E-07 | 2400 |
| neutral - threat | LTA | -0.1853706 | 0.02852699 | 3592 | -6.4980761 | 6.07E-09 | 2500 |
| neutral - threat | HTA | -0.154999 | 0.02746892 | 3592 | -5.6427053 | 1.14E-07 | 2500 |
| neutral - dysphoric | HTA | -0.1381605 | 0.02746892 | 3592 | -5.0297048 | 3.08E-06 | 2500 |
| neutral - threat | LTA | -0.1876449 | 0.02853501 | 3568 | -6.5759528 | 5.05E-09 | 2600 |
| neutral - threat | HTA | -0.1667932 | 0.02746966 | 3568 | -6.071908 | 1.31E-08 | 2600 |
| neutral - dysphoric | HTA | -0.1411926 | 0.02746966 | 3568 | -5.1399484 | 1.73E-06 | 2600 |
| neutral - threat | LTA | -0.1752942 | 0.02882656 | 3568 | -6.0809965 | 1.26E-08 | 2700 |
| neutral - threat | HTA | -0.1536603 | 0.02768822 | 3568 | -5.5496624 | 1.89E-07 | 2700 |
| neutral - threat | LTA | -0.171264 | 0.02848476 | 3592 | -6.0124791 | 1.76E-08 | 2800 |
| neutral - threat | HTA | -0.1278192 | 0.02761194 | 3592 | -4.6291299 | 2.26E-05 | 2800 |
| neutral - threat | LTA | -0.1800214 | 0.02855369 | 3568 | -6.3046639 | 6.66E-09 | 2900 |
| neutral - threat | LTA | -0.1559153 | 0.0286657 | 3568 | -5.4390881 | 3.47E-07 | 3000 |
| neutral - threat | LTA | -0.1475614 | 0.02942916 | 314.812896 | -5.0141199 | 5.30E-06 | 3100 |
| neutral - threat | LTA | -0.1426737 | 0.02988391 | 303.849596 | -4.7742648 | 1.66E-05 | 3200 |
| neutral - dysphoric | LTA | -0.1350129 | 0.02889927 | 3532 | -4.6718462 | 1.84E-05 | 4600 |
| neutral - dysphoric | LTA | -0.1339103 | 0.02887145 | 3568 | -4.6381579 | 2.16E-05 | 4700 |
| neutral - dysphoric | LTA | -0.1425034 | 0.02872958 | 3580 | -4.9601626 | 4.40E-06 | 4800 |
| neutral - dysphoric | LTA | -0.1277206 | 0.02860597 | 3580 | -4.464825 | 4.88E-05 | 4900 |
| positive - threat | LTA | 0.13674975 | 0.02984263 | 312.70493 | 4.58236275 | 3.93E-05 | 6400 |
| dysphoric - threat | LTA | 0.13473767 | 0.02984263 | 312.70493 | 4.51493975 | 5.30E-05 | 6400 |
| dysphoric - threat | LTA | 0.14429206 | 0.03021584 | 270.646064 | 4.77537745 | 1.74E-05 | 6600 |
| positive - threat | HTA | 0.13501234 | 0.0288751 | 310.704923 | 4.67573588 | 2.59E-05 | 7900 |
| positive - threat | HTA | 0.15380902 | 0.03049254 | 333.147018 | 5.04415197 | 4.47E-06 | 12700 |
| positive - threat | HTA | 0.14883713 | 0.0304304 | 326.160893 | 4.89106722 | 9.37E-06 | 12800 |
| positive - threat | HTA | 0.14613086 | 0.02876611 | 333.448682 | 5.07996515 | 3.75E-06 | 12900 |
| neutral - positive | HTA | -0.137894 | 0.02876611 | 333.448682 | -4.7936261 | 1.47E-05 | 12900 |
| positive - threat | HTA | 0.13798416 | 0.02914861 | 321.968739 | 4.73381554 | 1.96E-05 | 14100 |
| positive - threat | HTA | 0.15780033 | 0.02960535 | 324.109536 | 5.33012874 | 1.10E-06 | 14200 |
| positive - threat | HTA | 0.14815098 | 0.02929952 | 334.71249 | 5.05642995 | 4.20E-06 | 14300 |
| positive - threat | HTA | 0.14285465 | 0.02946953 | 323.922325 | 4.84753682 | 1.15E-05 | 14400 |
| positive - threat | HTA | 0.15677345 | 0.0294426 | 324.397291 | 5.32471505 | 1.13E-06 | 14500 |
| neutral - positive | LTA | -0.0802046 | 0.03102721 | 302.18408 | -2.5849753 | 0.04979275 | 14600 |
| positive - threat | HTA | 0.15776583 | 0.03036645 | 327.407421 | 5.1953994 | 2.15E-06 | 14600 |
| positive - threat | HTA | 0.14991506 | 0.03063311 | 327.587604 | 4.89389013 | 9.23E-06 | 14700 |
| positive - threat | HTA | 0.14082378 | 0.02839747 | 3484 | 4.95902538 | 4.43E-06 | 15000 |
| neutral - positive | HTA | -0.132397 | 0.02839747 | 3484 | -4.6622822 | 1.93E-05 | 15000 |
| positive - threat | HTA | 0.1688204 | 0.02810601 | 3500 | 6.00655768 | 1.53E-08 | 15100 |
| neutral - positive | HTA | -0.141168 | 0.02810601 | 3500 | -5.0226971 | 3.19E-06 | 15100 |
| positive - threat | HTA | 0.17013922 | 0.02872076 | 319.173713 | 5.92391167 | 4.87E-08 | 15200 |
| neutral - positive | HTA | -0.1331552 | 0.02872076 | 319.173713 | -4.6362024 | 3.06E-05 | 15200 |
| positive - threat | HTA | 0.16485113 | 0.02909969 | 319.484628 | 5.66504692 | 1.96E-07 | 15300 |
| positive - threat | HTA | 0.15995818 | 0.02839642 | 320.094079 | 5.63304054 | 2.32E-07 | 15400 |
| neutral - positive | HTA | -0.1287411 | 0.02839642 | 320.09408 | -4.5337104 | 4.84E-05 | 15400 |
| positive - threat | HTA | 0.15875592 | 0.0293096 | 321.871434 | 5.4165155 | 7.12E-07 | 15500 |
| neutral - positive | HTA | -0.1390441 | 0.0293096 | 321.871434 | -4.7439772 | 1.87E-05 | 15500 |
| positive - threat | HTA | 0.15328119 | 0.02920762 | 327.782199 | 5.24798687 | 1.65E-06 | 15600 |
| positive - threat | HTA | 0.1439893 | 0.02992887 | 327.468631 | 4.81104999 | 1.36E-05 | 15700 |
| positive - threat | HTA | 0.14860076 | 0.02942812 | 263.125955 | 5.04961849 | 4.93E-06 | 15800 |
| positive - threat | HTA | 0.13532789 | 0.02975315 | 326.271112 | 4.54835489 | 4.50E-05 | 15900 |
| positive - threat | HTA | 0.14793825 | 0.03163962 | 310.354234 | 4.67572716 | 2.59E-05 | 16600 |
| neutral - positive | HTA | -0.1433316 | 0.0306231 | 328.679086 | -4.6805049 | 2.48E-05 | 17600 |
| neutral - positive | HTA | -0.1428337 | 0.03039384 | 320.582115 | -4.699428 | 2.30E-05 | 17700 |
| neutral - positive | HTA | -0.1458895 | 0.0297748 | 325.888926 | -4.8997626 | 9.00E-06 | 19900 |
| positive - threat | LTA | 0.15676937 | 0.03067472 | 314.961354 | 5.11070216 | 3.32E-06 | 20100 |
| neutral - positive | HTA | -0.1377493 | 0.03003138 | 332.375077 | -4.5868463 | 3.77E-05 | 20100 |
| positive - threat | LTA | 0.15248312 | 0.03049261 | 316.453939 | 5.00065836 | 5.64E-06 | 20200 |
| neutral - positive | HTA | -0.1496569 | 0.02957315 | 328.355801 | -5.060565 | 4.15E-06 | 20200 |
| positive - threat | HTA | 0.1357666 | 0.02957315 | 328.355801 | 4.59087334 | 3.72E-05 | 20200 |
| positive - threat | LTA | 0.1363152 | 0.03016333 | 310.372055 | 4.51923599 | 5.21E-05 | 20300 |
| neutral - positive | HTA | -0.1424922 | 0.02908938 | 322.928189 | -4.898426 | 9.09E-06 | 20300 |
| positive - threat | HTA | 0.13616974 | 0.02908938 | 322.928189 | 4.68108124 | 2.49E-05 | 20300 |
| positive - threat | LTA | 0.14320212 | 0.03037666 | 291.791538 | 4.71421539 | 2.23E-05 | 20400 |
| neutral - positive | HTA | -0.1560921 | 0.0292004 | 299.735993 | -5.3455455 | 1.07E-06 | 20400 |
| positive - threat | LTA | 0.14298632 | 0.03148396 | 317.012169 | 4.54156022 | 4.69E-05 | 20500 |
| neutral - positive | HTA | -0.1462833 | 0.03043471 | 332.862084 | -4.8064633 | 1.38E-05 | 20500 |
| neutral - positive | HTA | -0.1547416 | 0.03106269 | 319.468088 | -4.9815883 | 6.15E-06 | 20600 |
| neutral - positive | HTA | -0.1695086 | 0.03144533 | 326.22116 | -5.3905805 | 8.06E-07 | 20700 |
| neutral - positive | HTA | -0.1571435 | 0.03117503 | 325.825837 | -5.040683 | 4.59E-06 | 20800 |
| positive - threat | LTA | 0.14843633 | 0.03152025 | 314.509768 | 4.70923729 | 2.21E-05 | 20900 |
| neutral - positive | HTA | -0.15522 | 0.03045642 | 324.185654 | -5.0964634 | 3.51E-06 | 20900 |
| positive - threat | HTA | 0.1390068 | 0.03045642 | 324.185655 | 4.56412165 | 4.21E-05 | 20900 |
| positive - threat | LTA | 0.16775545 | 0.03076031 | 314.043059 | 5.45363282 | 5.98E-07 | 21000 |
| neutral - positive | HTA | -0.1516013 | 0.02960094 | 321.404249 | -5.121502 | 3.12E-06 | 21000 |
| positive - threat | HTA | 0.14866441 | 0.02960094 | 321.404249 | 5.02228721 | 5.05E-06 | 21000 |
| positive - threat | LTA | 0.1567495 | 0.0317422 | 322.548858 | 4.93820487 | 7.53E-06 | 21100 |
| neutral - positive | HTA | -0.1613808 | 0.03038342 | 325.69912 | -5.3114756 | 1.21E-06 | 21100 |
| positive - threat | HTA | 0.15371332 | 0.03038342 | 325.69912 | 5.05911785 | 4.20E-06 | 21100 |
| positive - threat | LTA | 0.15556081 | 0.0312503 | 326.081112 | 4.97789865 | 6.20E-06 | 21200 |
| neutral - positive | HTA | -0.1543894 | 0.03002137 | 330.774973 | -5.1426479 | 2.77E-06 | 21200 |
| positive - threat | HTA | 0.14986776 | 0.03002137 | 330.774973 | 4.99203518 | 5.76E-06 | 21200 |
| positive - threat | LTA | 0.15942131 | 0.0308001 | 330.022554 | 5.17599951 | 2.35E-06 | 21300 |
| neutral - positive | HTA | -0.1511989 | 0.02941188 | 324.852156 | -5.140743 | 2.82E-06 | 21300 |
| positive - threat | HTA | 0.15016399 | 0.02941188 | 324.852156 | 5.10555535 | 3.35E-06 | 21300 |
| neutral - positive | HTA | -0.1517193 | 0.03065513 | 324.728665 | -4.9492301 | 7.13E-06 | 21400 |
| positive - threat | HTA | 0.14702178 | 0.03065513 | 324.728665 | 4.79599211 | 1.47E-05 | 21400 |
| positive - threat | HTA | 0.15467427 | 0.0306422 | 320.267131 | 5.04775349 | 4.47E-06 | 21500 |
| neutral - positive | HTA | -0.1535397 | 0.0306422 | 320.267131 | -5.0107267 | 5.35E-06 | 21500 |
| neutral - positive | HTA | -0.1576724 | 0.03102959 | 330.682254 | -5.0813555 | 3.74E-06 | 21600 |
| positive - threat | HTA | 0.15066406 | 0.03102959 | 330.682254 | 4.85549673 | 1.10E-05 | 21600 |
| neutral - positive | HTA | -0.1449894 | 0.0293648 | 338.357461 | -4.9375244 | 7.40E-06 | 21700 |
| positive - threat | HTA | 0.1360419 | 0.0293648 | 338.357461 | 4.63282187 | 3.05E-05 | 21700 |
| neutral - positive | HTA | -0.1622464 | 0.02858465 | 324.744604 | -5.6759965 | 1.83E-07 | 21800 |
| positive - threat | HTA | 0.14696512 | 0.02858465 | 324.744604 | 5.14139961 | 2.81E-06 | 21800 |
| neutral - positive | HTA | -0.1775117 | 0.0287942 | 320.824698 | -6.1648433 | 1.27E-08 | 21900 |
| positive - threat | HTA | 0.16220441 | 0.0287942 | 320.824698 | 5.63323301 | 2.31E-07 | 21900 |
| neutral - positive | HTA | -0.1869937 | 0.02918388 | 356.388416 | -6.4074311 | 2.81E-09 | 22000 |
| positive - threat | HTA | 0.17590146 | 0.02918388 | 356.388416 | 6.02735118 | 2.49E-08 | 22000 |
| neutral - positive | HTA | -0.1744482 | 0.0282943 | 323.315604 | -6.1654908 | 1.25E-08 | 22100 |
| positive - threat | HTA | 0.17419779 | 0.0282943 | 323.315604 | 6.15663971 | 1.32E-08 | 22100 |
| neutral - positive | HTA | -0.1658913 | 0.02906216 | 320.940214 | -5.7081527 | 1.56E-07 | 22200 |
| positive - threat | HTA | 0.1588704 | 0.02906216 | 320.940214 | 5.46657167 | 5.52E-07 | 22200 |
| positive - threat | HTA | 0.18547261 | 0.02895141 | 329.648014 | 6.40634099 | 3.08E-09 | 22300 |
| neutral - positive | HTA | -0.1577017 | 0.02895141 | 329.648014 | -5.4471155 | 5.99E-07 | 22300 |
| positive - threat | HTA | 0.19528769 | 0.02929611 | 300.560928 | 6.66599321 | 7.55E-10 | 22400 |
| neutral - positive | HTA | -0.1519149 | 0.02929611 | 300.560928 | -5.1854965 | 2.36E-06 | 22400 |
| positive - dysphoric | HTA | 0.13810784 | 0.02929611 | 300.560928 | 4.71420355 | 2.20E-05 | 22400 |
| positive - threat | HTA | 0.2007211 | 0.02995883 | 328.710929 | 6.69989687 | 5.44E-10 | 22500 |
| neutral - positive | HTA | -0.1475033 | 0.02995883 | 328.710929 | -4.9235327 | 8.01E-06 | 22500 |
| positive - dysphoric | HTA | 0.13726322 | 0.02995883 | 328.710929 | 4.58172762 | 3.87E-05 | 22500 |
| positive - threat | HTA | 0.19922049 | 0.02922102 | 318.92495 | 6.81771264 | 2.79E-10 | 22600 |
| positive - threat | HTA | 0.20385154 | 0.02952873 | 318.966589 | 6.9034974 | 1.66E-10 | 22700 |
| positive - threat | HTA | 0.18683006 | 0.03008598 | 327.111271 | 6.20987047 | 9.64E-09 | 22800 |
| positive - threat | HTA | 0.16449087 | 0.03022156 | 324.811173 | 5.44283221 | 6.18E-07 | 22900 |
| positive - threat | HTA | 0.14969183 | 0.03032202 | 319.527921 | 4.93673751 | 7.62E-06 | 23000 |
| positive - threat | HTA | 0.14190023 | 0.02989579 | 320.223606 | 4.74649601 | 1.85E-05 | 23100 |
| positive - threat | HTA | 0.15768457 | 0.02929042 | 319.680699 | 5.38348569 | 8.46E-07 | 23200 |
| positive - threat | HTA | 0.16891686 | 0.02953815 | 324.367 | 5.71859946 | 1.46E-07 | 23300 |
| positive - threat | HTA | 0.16875421 | 0.03031543 | 318.253489 | 5.56661031 | 3.30E-07 | 23400 |
| positive - threat | HTA | 0.16885704 | 0.03050872 | 322.715539 | 5.53471423 | 3.86E-07 | 23500 |
| positive - threat | HTA | 0.15614198 | 0.03095673 | 325.349331 | 5.04387901 | 4.52E-06 | 23600 |
| positive - threat | HTA | 0.13935296 | 0.03059661 | 325.274298 | 4.55452336 | 4.39E-05 | 23700 |
| positive - threat | HTA | 0.16253469 | 0.0313591 | 322.849739 | 5.18301464 | 2.30E-06 | 23800 |
| positive - threat | LTA | 0.14038237 | 0.0307175 | 302.298888 | 4.57010997 | 4.20E-05 | 23900 |
| positive - threat | HTA | 0.14689922 | 0.02979908 | 319.226491 | 4.92965631 | 7.88E-06 | 23900 |
| positive - threat | LTA | 0.1370939 | 0.03035948 | 303.853009 | 4.5156866 | 5.33E-05 | 24000 |
| positive - threat | HTA | 0.14371361 | 0.02930674 | 314.072225 | 4.90377302 | 8.98E-06 | 24000 |
| positive - threat | LTA | 0.1541488 | 0.03099122 | 305.40075 | 4.97395041 | 6.52E-06 | 24200 |
| positive - threat | HTA | 0.1372444 | 0.02993996 | 316.092491 | 4.58398757 | 3.88E-05 | 24200 |
| positive - threat | LTA | 0.1620354 | 0.03178153 | 306.610913 | 5.09841372 | 3.58E-06 | 24300 |
| positive - threat | LTA | 0.16978559 | 0.03229572 | 303.019837 | 5.25721615 | 1.65E-06 | 24400 |
| positive - threat | LTA | 0.19454732 | 0.03292024 | 309.578347 | 5.90965655 | 5.41E-08 | 24500 |
| positive - threat | LTA | 0.1666449 | 0.03281122 | 305.3584 | 5.07890027 | 3.94E-06 | 24600 |
| positive - threat | LTA | 0.15134842 | 0.03122884 | 202.015358 | 4.84643066 | 1.48E-05 | 24700 |
| positive - threat | HTA | 0.13799035 | 0.02999321 | 202.405867 | 4.60072008 | 4.37E-05 | 24700 |
| positive - threat | LTA | 0.16106558 | 0.0344944 | 314.596869 | 4.66932588 | 2.65E-05 | 24800 |
| positive - threat | LTA | 0.15354462 | 0.03338757 | 323.834551 | 4.59885641 | 3.60E-05 | 25000 |
| positive - threat | HTA | 0.14611135 | 0.03206332 | 326.978939 | 4.55696297 | 4.33E-05 | 25000 |
| positive - threat | HTA | 0.1448989 | 0.03141409 | 327.45199 | 4.61254495 | 3.38E-05 | 25100 |
| positive - threat | HTA | 0.15464324 | 0.03228727 | 326.842369 | 4.78960343 | 1.51E-05 | 25200 |
| positive - threat | HTA | 0.15507854 | 0.03194529 | 324.215625 | 4.8545045 | 1.12E-05 | 25300 |
| neutral - positive | HTA | -0.144886 | 0.03158151 | 328.611065 | -4.5876858 | 3.77E-05 | 26000 |
| positive - dysphoric | LTA | 0.15780043 | 0.03236464 | 319.66362 | 4.87570448 | 1.02E-05 | 26600 |
| neutral - positive | HTA | -0.1482296 | 0.03099842 | 327.203001 | -4.7818433 | 1.56E-05 | 26800 |
| positive - threat | HTA | 0.14173661 | 0.03099842 | 327.203001 | 4.5723812 | 4.04E-05 | 26800 |
| neutral - positive | HTA | -0.1376378 | 0.0302596 | 330.244632 | -4.5485661 | 4.48E-05 | 27000 |
| positive - threat | HTA | 0.14758782 | 0.03082209 | 330.836697 | 4.78837777 | 1.51E-05 | 27100 |
| positive - threat | HTA | 0.16693797 | 0.03013009 | 320.399189 | 5.5405731 | 3.76E-07 | 27200 |
| neutral - positive | HTA | -0.1556794 | 0.03013009 | 320.399189 | -5.1669093 | 2.50E-06 | 27200 |
| positive - threat | HTA | 0.17919381 | 0.03025867 | 323.329522 | 5.92206524 | 4.87E-08 | 27300 |
| neutral - positive | HTA | -0.1427432 | 0.03025867 | 323.329522 | -4.7174301 | 2.11E-05 | 27300 |
| positive - threat | HTA | 0.16789872 | 0.03031952 | 322.885246 | 5.53764475 | 3.80E-07 | 27400 |
| positive - threat | HTA | 0.16635231 | 0.0296002 | 187.00763 | 5.61997292 | 4.09E-07 | 27500 |
| positive - threat | HTA | 0.15891783 | 0.03149535 | 331.508015 | 5.04575609 | 4.44E-06 | 27600 |
| positive - threat | HTA | 0.16809437 | 0.03130208 | 328.718472 | 5.37007048 | 8.91E-07 | 27700 |
| positive - threat | HTA | 0.15728905 | 0.03207096 | 328.22952 | 4.90440778 | 8.78E-06 | 27800 |
| positive - threat | HTA | 0.14669623 | 0.03117797 | 320.154467 | 4.70512511 | 2.24E-05 | 29400 |
